# Percutaneous endoscopic debridement with percutaneous pedicle screw fixation for lumbar pyogenic spondylodiscitis: a preliminary study

**DOI:** 10.1007/s00264-019-04456-1

**Published:** 2019-12-26

**Authors:** Kaidi Duan, Yi Qin, Jichao Ye, Wei Zhang, Xumin Hu, Jinlang Zhou, Liangbin Gao, Yong Tang

**Affiliations:** 1 Dept. of Spine Surgery, Sun Yat-sen Memorial Hospital of Sun Yat-sen University, 107 Yanjiang West Road, Guangzhou, China; 2 Dept. of Orthopedics, Zhuhai People’s Hospital, 79 Kangning Road, Zhuhai, China; 3 Dept. of Orthopedics, Xinsteel Center Hospital, Tuanjie West Road, Xinyu, China

**Keywords:** Spinal infection, Spondylodiscitis, Debridement, Percutaneous pedicle screw fixation, Percutaneous spine endoscopy

## Abstract

**Purpose:**

To access the feasibility and efficacy of percutaneous endoscopic debridement (PED) combined with percutaneous pedicle screw fixation (PPSF) in the treatment of lumbar pyogenic spondylodiscitis.

**Methods:**

Forty-five patients diagnosed as pyogenic spondylodiscitis underwent PPSF followed by PED. A drainage catheter was left in place for negative pressure drainage. Adequate systematic antibiotics were administered empirically or based on bacterial culture results. Clinical outcomes were assessed by physical examination, regular serologic testing, visual analog scale (VAS), Oswestry Disability Index (ODI), and imaging studies.

**Results:**

The mean operative time was 110.1 ± 21.2 minutes (range 80–165 minutes), with intra-operative blood loss 47.8 ± 21.0 ml (range 20–120 ml). All patients reported relief of back pain, able to sit up, and partially ambulate the next day. Causative pathogens were identified in 32 of 45 biopsy specimens, staphylococcal bacteria being the most prevalent strain. However, there were 13 patients with post-operative complications. During 6–12 months’ follow-up, inflammatory markers showed infection controlled. VAS and ODI values were significantly improved.

**Discussion:**

Satisfactory clinical and functional outcomes were achieved in our patients post-operatively. It is recommended that PED plus PPSF can be another alternative for spondylodiscitis.

**Conclusion:**

PED supplementing PPSF offers a valid option in treating spondylodiscitis, as it is minimally invasive, shortens hospital stay, and avoids prolonged bed rest with an optimistic outcome.

## Introduction

Spondylodiscitis is the most commonly seen pyogenic spinal infection that affects intervertebral disc, adjacent vertebrae, and surrounding structures [1]. By the end of nineteenth century, according to Makins and Abbot’s study, the reported death rate of pyogenic spondylitis in children and other young patients could reach 70% [2]. A significant change in the prognosis of the affected children was then achieved with the use of antibiotics [3]. The proportion of thoraco-lumbar spondylitis accounts for over 80% of vertebral infection [4]. Surgical intervention is typically reserved for patients in case of unresponsive antibiotic treatment, severe kyphotic deformity or progressive instability of vertebral column, epidural abscess, and significant neurological deficit [5]. With the progress in surgical techniques and the development of internal fixation implants, active surgical managements reduce hospital stay and avoid prolonged bed rest; thus, its surgical indication broadens for the treatment of pyogenic spondylitis.

As a classic surgical approach, anterior debridement followed by posterior internal fixation offers definitive treatment for lumbar pyogenic spondylitis [6, 7]. Its downsides include massive surgical trauma, frequent incidence of peri-operative complications, and relatively high mortality rate among the elders and the vulnerable patients. Single-stage debridement through a posterior approach for vertebral decompression and instrumentation is used to apply to cases with abscess formation in the vertebral canal [8] as well as increasingly being favoured in the treatment of infectious spondylitis recently [9]. But it has also been questioned by some scholars due to its structural damage and risk of infection spreading to the posterior column. As the elders tend to be more susceptible to the pyogenic spondylitis, higher risk of complications and mortality brought by conventional open surgery is the undesired consequence surgeons have to face [10].

In recent years, miscellaneous minimally invasive surgical techniques have targeted this ailment [11, 12] and proved to be advantageous. Percutaneous endoscopic debridement that serves as a diagnostic and therapeutic technique has been increasingly accepted and combined with other surgical practices. From January 2014 to December 2016, we successfully treated 45 cases of lumbar pyogenic spondylodiscitis via percutaneous endoscopic debridement (PED) following percutaneous pedicle screw fixation (PPSF). The purpose of this study was to evaluate the feasibility and efficacy of PED supplementing PPSF in the treatment for lumbar pyogenic spondylodiscitis.

## Clinical materials and methods

From January 2014 to December 2016, 45 patients (27 male and 18 female patients) diagnosed as pyogenic spondylodiscitis were retrospectively enrolled into our study. Their major symptoms presented as intractable immobilizing pain in the lower back that worsened at night, fever, and other unspecific infection signs. Progressive septicemia was ruled out. In a few patients, aggravating neurologic deficits of the bladder and limbs were noted, and prompt surgical management was proceeded.

The laboratory chemical parameters that help confirm the diagnosis of vertebral infection include erythrocyte sedimentation rate (ESR), elevated C-reactive protein (CRP), microbiological culture results along with radiological signs in X-ray, computed tomography (CT) scans, and magnetic resonance imaging (MRI) findings. Our inclusion criteria for surgery are of patients with the following: (1) intolerable back and/or radiating pain caused by infectious spondylitis that cannot be managed by conservative treatment; (2) deteriorating muscle strength of the limbs and other neurological damage; (3) lumbar instability with osseous lesions; (5) epidural/paravertebral abscess formation.

The following is to be excluded: (1) giant para-vertebral abscess; (2) severely damaged vertebral body (more than 1/3 vertebral body compromised); (3) signs of cauda equina syndrome due to epidural abscess; (4) nerve compression by abscess formed in the dorsal side of dura mater; (5) specific pathogens such as *Mycobacterium tuberculosis* and *Brucella*.

After ruling out contraindications and obtaining written and informed consent, PED with PPSF was subsequently performed.

### Operative procedure

Under general anaesthesia, patients were positioned prone on the U-shaped cushion with abdomen free. Neural evoked potential detector at ready in case of necessity. The surgery consisted of two steps: percutaneous pedicle screw fixation usually prior to percutaneous endoscopic debridement.

First, mark the target vertebral pedicles and intervertebral space under fluoroscopic guidance. A small stab incision was made for each screw at the level to be fused. A vertebroplasty needle was inserted toward the pedicle and vertebral body before replaced by a K-wire. Soft tissue dilation along the K-wire and pedicle tapping was then achieved. Next screws were advanced along the prepared passage. The length and direction of the pedicle screw were planned pre-operatively and adjusted intra-operatively. Finally, pedicle rods were passed through the relevant trajectory of screws.

For endoscopic debridement, Yeung’s technique was selected to get access to the target site for debriding. An 18-gauged long needle was directed to the target intervertebral space to collect the fluid sample. In case of sample scarcity, 10–20 ml sterile saline can be injected and then aspirated. The sample was collected for mircrobiological culture (both aerobic and anaerobic). A guide wire was introduced into the disc space through the spine needle which was then withdrawn. After making a 1-cm cut, a dilator and a cannulated sleeve were subsequently inserted to the disc space. In case of spine canal abscess, the tip of dilator should be placed at the foyer to facilitate the opening, washout, and drainage of the abscess. In this situation, spontaneous electromyography and evoked potential monitoring are required to avoid possible nerve root damage during the intracanal procedure of this kind. Correct placement of the sleeve should be verified by fluoroscopic images on two orthogonal planes before removing the dilator and introducing the endoscope. Debridement was performed piecemeal by various endoscopic tools in order to remove and clean out necrotic tissues as much as possible, which in turn lowers tension of the discs. More than six lites normal saline is suggested for effective irrigation of the disc space to wash out the remaining abscess and necrotic tissue. Before closing up, a hard tube was placed at the debrided disc space and connected to a negative pressure device for continuous drainage.

### Post-operative management

The infected tissue samples were sent for bacterial culture and pathological analysis. All the tubes were left in place until the drainage stopped, usually in the following seven to 14 days. When the pain shows alleviation, early mobilization and exercise of back and waist muscles should be encouraged as it reduces amyotrophy and promotes overall recovery.

Effective antibiotics, generally three to four weeks intravenously and six to eight weeks orally taken, are to be administered timely according to the bacterial culture results. If the result is histopathologically positive for pyogenic infection but negative from bacterial culture, the antibiotic regime of choice should cover commonly seen gram negative and positive bacteria, for example, vancomycin with levofloxacin. These dual broad-spectrum intravenous antibiotics are also empirically used before the surgery or the delivery of bacterial resistance reports once the infection is highly suspected.

To keep track of infection control post-operatively, laboratory chemical parameters, including ESR, CRP, and a routine blood count, were checked weekly and recorded till they dropped to the normal base. Apart from the tests mentioned above, antero-posterior and lateral plain films are needed during one, three, six, 12, and 24 months follow-ups. When necessary, MRI (enhanced MRI included) should also be taken into consideration.

Pre-operative state and post-operative clinical outcomes were assessed by visual analog scale (VAS) for lumbar pain, Oswestry Disability Index (ODI) as functional outcome criteria, and the neurologic state was evaluated according to the American Spinal Injury Association (ASIA) impairment scale. Post-operative complications were recorded during the follow-up period.

## Results

From January 2014 to December 2016, 45 patients with a mean age of 51.2 ± 14.6 years (6 patients with L1/2 disc infection, 8 with L2/3, 11 with L3/4, 10 with L4/5, 1 with L1–3, 4 with L5/S1, 2 with L2–4, 2 with L3–5, 1 with L4-S1) from three different co-operated hospitals were retrospectively enrolled into the study and subsequently received the PED with PPSF procedure as described.

Upon admission, these 45 patients presented with an average VAS of 7.5 ± 0.9 and an ODI (%) of 78.6 ± 9.4. Laboratory results demonstrated mean baseline CRP serum level concentrations of 62.6 ± 38.7 mg/L, mean ESR level of 90.8 ± 37.9 mm/hour, and elevated leucocyte with an average of (14.0 ± 4.1) × 10^9^/L. Twenty-seven patients were of primary infection, while eight were of post-operative infection from invasive procedures of spine and one from urinary tract procedure. Systemic sepsis, pneumonia, and other causes of infection in recent three months consisted of the remaining ten cases (Table 1). Recent bacterial infection, diabetes mellitus, and cardio-related diseases were the most common comorbidities observed in our subjects.

**Table 1:** Cause of infection (*n* patients)

Primary infection of unknown cause	27
Invasive lumbar procedures	
Percutaneous endoscopic lumbar discectomy	3
Open lumbar discectomy	2
Vertebral disc puncture	1
Radiofrequency ablation	2
Urinary tract procedure	1
Systemic sepsis	3
Pneumonia	2
Superficial/deep soft tissue infection	3
Deep abdominal infection	1

The mean operative time was 110.1 ± 21.2 minutes (range 80–165 minutes), with intra-operative blood loss 47.8 ± 21.0 ml (range 20–120 ml). As the most complaining symptom of pyogenic spondylodiscitis, low back pain was resolved immediately after the operation, with partial ambulation on bed and sleep improvement at night. Limited range of motion (ROM) could be initiated two to three days after the surgery under the protection of elastic waist strap.

Thirteen patients showed signs of surgery-related complications, notably seven with secondary infection, one with loosening of implants, and two with radiculopathy (ASIA D). Two patients had evident neurologic impairment: one patient experienced lumbar radiculopathy with degradation of muscle strength by one degree; the other reported to have numbness in the lower limb. Their symptoms both occurred on the ipsilateral side of surgical site. Advanced age with multicomorbidities (diabetes, osteoporosis, chronic pulmonopathy, etc.) might have resulted in slower response to the therapy and the susceptibility to infection. In an inflamed and affected state preoperatively, the nerve root could less tolerate the irritation from sleeve building and other neighbouring surgical procedures, which might be the major cause of neurological impairment.

Overall, causative pathogens were identified in 32 cases (Table 2) with the most common being staphylpcoccal bacteria. Microbiological analyses of specimens showed that five samples were *Staphylococcus aureus* positive, seven *Staphylococcus epidermidis* positive, five *Escherichia coli* positive, three *Klebsiella pneumoniae* positive, and the rest identified other pathogens. Likely due to the pre-operative use of antibiotics, 13 samples were negative in bacterial culture but pathologically positive with septic inflammatory change.

**Table 2:** Pathogens detected (*n* patients)

*Staphylococcus aureus*	5
*Staphylococcus epidermidis*	7
*Enterococcus faecalis*	2
*Escherichia coli*	5
*Klebsiella pneumoniae*	3
Others	10
None identified	13

**Fig. 1:**
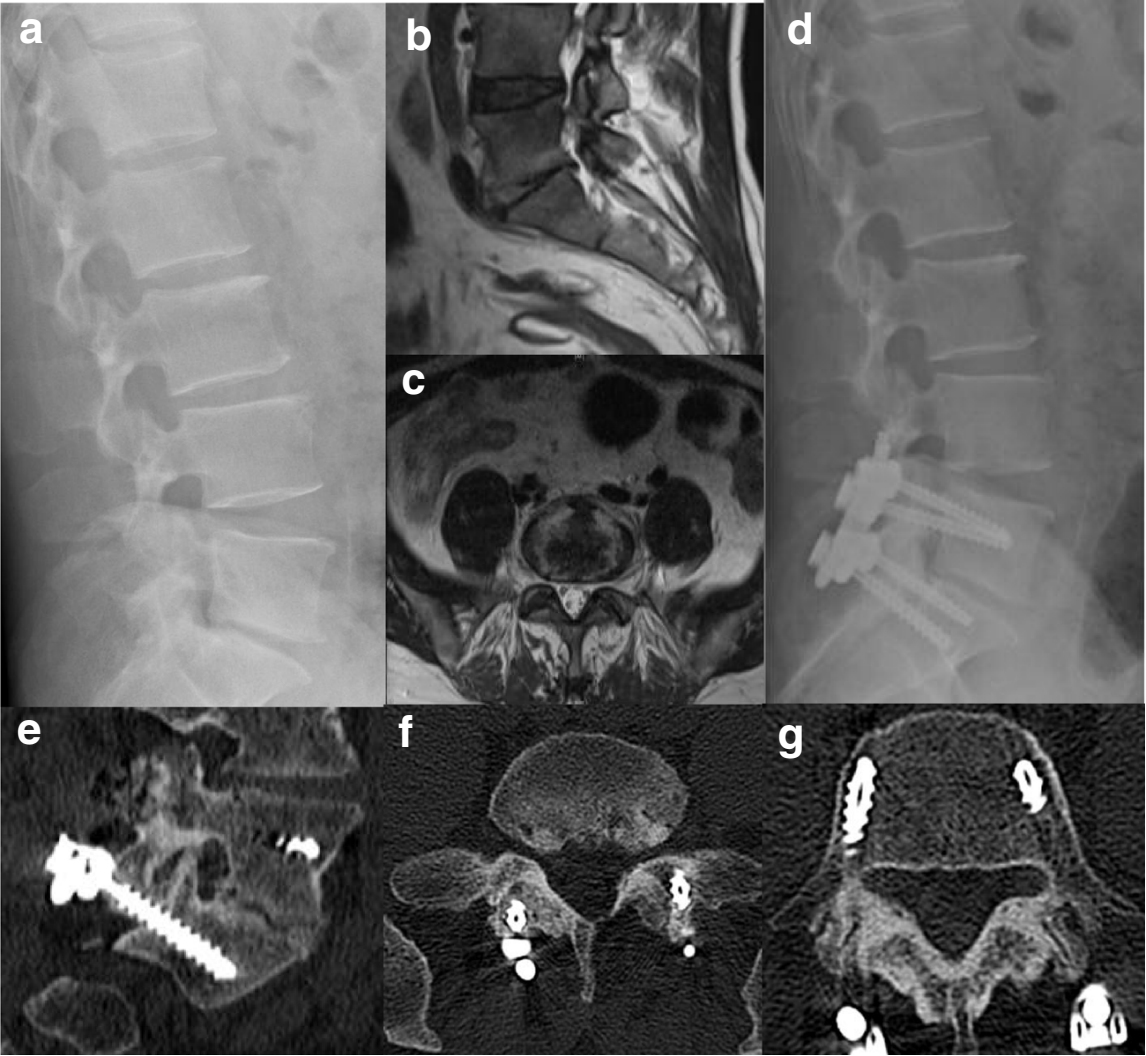
Exemplary case of a 47-year-old male presenting with immobilizing low back pain (VAS 5/10), fever and elevated procalcitonin, ESR, and CRP level. X-ray upon admission showed narrowing L5-S1 disc space with endplate destruction (**a**). Sagittal (**b**) and axial (**c**) T2-weighted MR images demonstrated bony endplate lesion and purulent mass at the L5/S1 level. Post-operative X-ray at 3 months (**d**) and spine computed tomography demonstrated bony fusion on the sagittal view at 33 months post-operatively (**e**). The canal was adequately debrided and decompressed on the axial view (**f**) with no screw loosening (**g**)

**Fig. 2:**
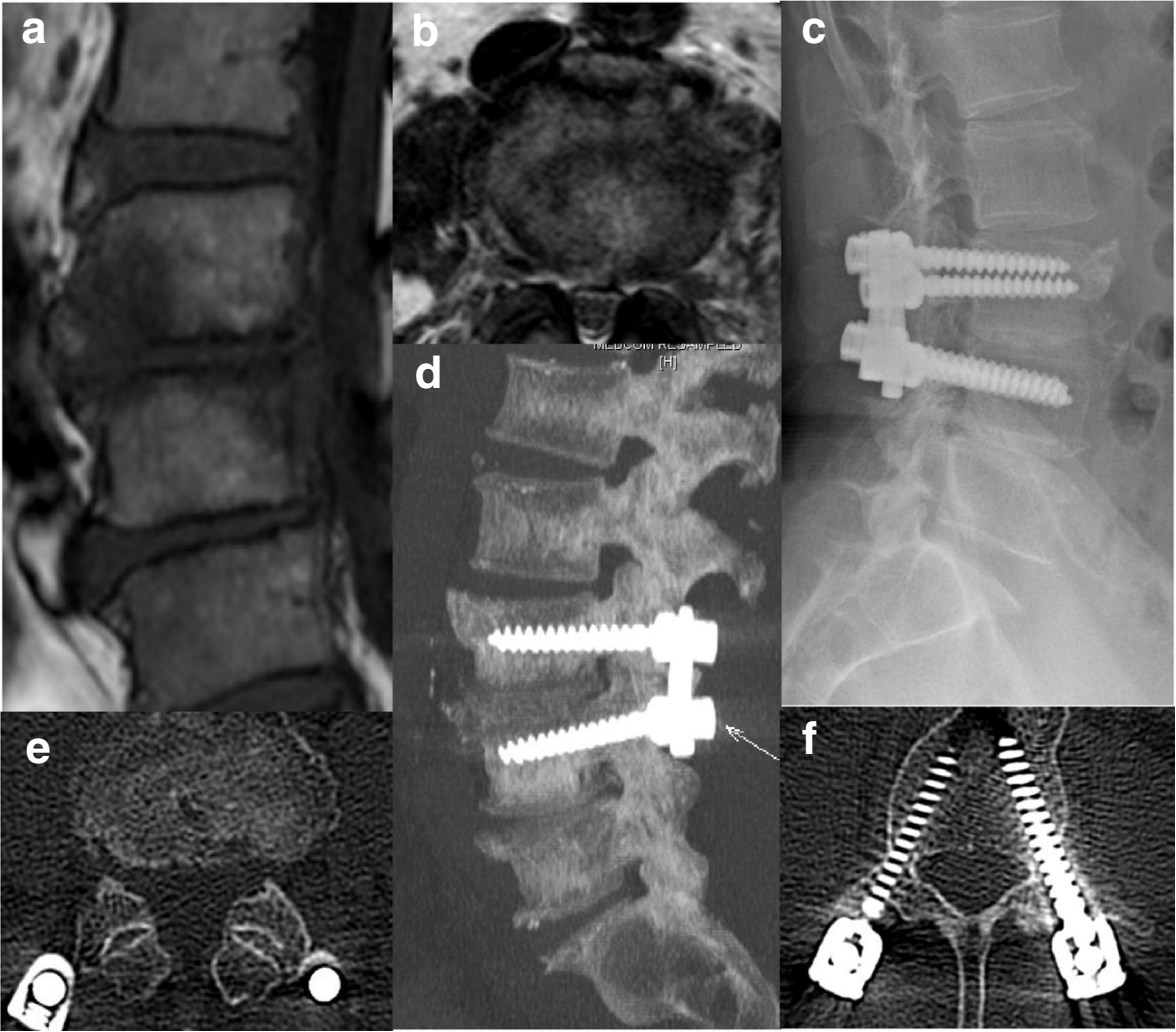
Exemplary case of a 61-year-old female presenting with immobilizing low back pain (VAS 6/10), fever and elevated procalcitonin, ESR, and CRP level. Sagittal (**a**) T1-weighted showed abscess in the frontal part of L3/4 disc space and axial (**b**) T2-weighted MR images demonstrated bony endplate lesion and purulent mass at the same level. Post-operative X-ray at 12 months (**c**) and spine computed tomography demonstrated bony fusion on the sagittal view at 30 months post-operatively (**d**). The canal was adequately debrided and decompressed on the axial view (**e**) with no screw loosening (**f**)

During six to 24 months of post-operative follow-up, the mean VAS decreased to 0.5 with an average ODI (%) of 14.5 ± 9.3. For most patients, the CRP levels, WBC normalized within three months after operation while less than half of them showed slightly elevated ESR (Table 3). Two cases observed no obvious decrease in inflammatory markers: one (L3-5 infection, *Escherichia coli*) who experienced epidermal infection of the wound, received intervertebral debridement and autogenous iliac bone graft through anterior approach three months after the initial operation; the other (L2/3 infection) was *Mycobacterium chelonae* positive in his microbiolgcal culture. Change of antibiotic regimen and prolonged therapy managed to keep the infection under control half a year later. In the long term, the debrided disc space would form bony structures to achieve solid interbody fusion and satisfactory stability (Figs. 1 and 2), which demonstrates a consistently promising outcome.

**Table 3:** Pre-operative and post-operative follow-up CRP, ESR, WBC, ODI, VAS, and ASIA grade

Case no.	CRP (mg/L)		ESR (mm/h)		WBC (× 10^9^/L)		ODI		VAS score		ASIA grade	
	Pre-op	3 months	Pre-op	3 months	Pre-op	3 months	Pre-op	Final	Pre-op	Final	Pre-op	Final
1	120	15	156	25	18.4	5.7	88	14	9	0	E	E
2	42	20	30	10	12.5	6.7	78	8	8	1	E	E
3	68	< 5	90	12	10.8	4.9	72	16	8	0	E	E
4	59	18	95	15	15.6	8.8	76	14	7	1	E	E
5	34	< 5	49	15	16.5	6.2	84	22	8	2	D	E
6	42	18	95	30	12.3	5.7	82	8	6	0	C	D
7	60	< 5	150	25	14.5	5.7	92	10	9	1	E	E
8	27	15	66	10	9.8	8.1	90	14	8	0	E	E
9	85	< 5	135	18	11.5	5.1	70	16	8	0	E	E
10	130	< 5	120	25	6.8	6.8	74	8	7	0	D	E
11	225	90	200	120	16.5	11.1	76	46	7	3	E	E
12	50	21	120	40	15.4	7	91	10	8	1	E	E
13	144	< 5	95	8	19.8	6.9	92	22	7	0	D	D
14	39	17	85	15	8.4	6.9	76	20	8	1	E	D
15	67	< 5	190	12	8.8	5.4	72	14	7	0	E	E
16	84	18	64	26	6.7	5	68	20	8	0	E	E
17	57	< 5	55	24	12.8	6.1	59	6	8	0	E	E
18	60	54	94	105	19.8	9.4	66	54	7	4	D	E
19	76	< 5	97	21	22.4	9	92	6	8	0	E	E
20	59	< 5	67	22	10.8	5.7	70	12	7	1	E	E
21	81	< 5	94	19	12.7	6.9	68	18	9	0	E	E
22	39	12	68	10	8.5	5.4	92	22	7	1	E	E
23	27	< 5	55	18	6.7	8.7	88	16	7	0	D	E
24	15	8	64	30	13.4	4.9	82	10	7	1	E	E
25	99	< 5	137	25	21.7	6.7	68	6	8	0	E	E
26	15	7	60	8	17.4	6.4	90	14	8	1	E	E
27	68	11	68	19	16.1	8.8	88	16	8	0	D	D
28	< 5	< 5	34	14	17.2	7.9	84	8	7	0	E	E
29	67	6	97	15	13.9	6.7	84	10	8	1	E	E
30	38	< 5	68	8	12.1	5.4	58	8	7	0	E	E
31	91	19	64	10	20.1	9.1	90	14	7	0	D	E
32	45	< 5	56	11	13.5	6.7	68	16	6	1	E	E
33	48	10	67	6	11.5	8.1	82	8	7	0	E	E
34	105	< 5	99	9	12.5	5.4	76	22	9	2	E	D
35	64	< 5	151	19	15.8	6.2	72	14	8	0	E	E
36	40	< 5	132	17	16.4	5.3	68	6	5	0	E	E
37	27	< 5	67	21	15.9	6.4	74	10	8	1	D	E
38	38	< 5	94	16	19.9	8.1	78	4	7	0	E	E
39	44	8	83	25	15.6	9.7	84	8	8	0	E	E
40	50	7	67	18	9.7	5.1	88	16	9	0	E	E
41	79	< 5	94	30	10.4	6.8	82	20	5	2	E	E
42	67	< 5	108	28	16.2	3.9	84	12	7	0	D	E
43	53	< 5	65	15	15.9	6.4	70	8	7	0	E	E
44	31	11	54	6	10	6.4	68	16	8	1	E	E
45	57	< 5	87	14	18.1	8.2	82	10	7	0	E	E

## Discussion

Reportedly, over 60% affecting the lumbar region, pyogenic spondylodiscitis is one of the most severe, specific pathologies with poor prognosis which needs prompt diagnosis and appropriate antibiotic treatment [13]. In recent years, the elevated rate of vertebral pyogenic infection has been attributed to compromised immune state, the aging of population, iatrogenically invasive procedures, the increasing comorbidities of all systems, improved diagnostic possibilities, etc. [14, 15]. As a major ailment, pyogenic spondylitis eventually causes severely limited function as well as immobilizing back pain of varied degrees and distressing symptoms of lower limbs. Therefore, the objective of any treatment ought to aim at rapidly relieving the symptoms, improving lumbar stability, and helping the patients get their life and work back on track. Conservative treatment, open surgery, and minimally invasive surgery are among multiple candidate therapies addressing pyogenic spondylitis. Conservative treatment takes eight to 12 weeks of adequate antibiotic use as one complete course with bed rest and/or orthosis use. Over the following three months or longer, the affected vertebrae would repair and fuse by themselves until the bony fusion confines spinal movement and progressively alleviates the pain. This period could be rather distressing for patients and their family alike, especially for the elders with multiple comorbidities. Surgical intervention is indicted in the context of failed non-surgical treatment, aggravating radiculopathy and the need of biopsy extracting. Empirically speaking, we think to seize the optimal timing and prevent deterioration of the infection; surgery should be considered when clinical signs, lab, and imaging results show no improvement after two weeks of conservative treatment. Open surgery, otherwise, poses a second strike for patients due to massive iatrogenic trauma, possible spinal cord and nerve damage.

Yang et al. [11] gained satisfactory therapeutic effect in treating septic spondylitis via spine endoscope, but bed rest time was not reported and two patients eventually converted to open surgery due to the lasting pain. Although this surgical approach brings little damage to the tissue and posterior structure, the pain resulting from the debridement along with pre-existed anterior vertebral body damage still immobilized the patients on bed for a long time, which could be a disastrous outcome for the elders. When thorough posterior debridement and appropriate antibiotics can be guaranteed, extra instrumentation of pedicular screws or placement of metallic implants proves to offer better stability and fusion outcome without taking its toll on infection control, thus avoiding the need for anterior surgery and prolonged bed rest [16]. Some studies indicated that PPSF might be the candidate surgery for pyogenic spondylitis [17, 18], while others reported about its limitation [19]. Lin et al. [20] compared PPSF with open surgery and asserted advantages of PPSF with regard to lower blood loss, shorter time of surgery, less post-operative pain, and no negative impact on infection control. Forty-five patients of our group underwent PPSF followed by PED. They were able to sit up the next day and showed good physical functioning after the drainage tubes were removed. VAS scored ≤ 1 3 months later, and MacNab reached 100% at the ten months follow-up. Efficacious, abundant, full course antibiotic administration plays a vital role in the treatment for lumbar spondylodiscitis. Previously, 36–76% samples are positive in finding pathogens from bacteria culture [21, 22]. Fouquet et al. [23] reported 9 out of 25 samples (36%) confirmed bacterial infection using Mazabraud trocar for biopsy. Staatz [22] found 16 out of 21 patients’ samples (76%) obtained by CT-guided percutaneous catheter drainage-identified pathogens. Yang et al. [11] reported similar results by claiming 86.7% (13/15) bacterial cultures positive via spine endoscopic technique. In our study, pathogens were identified in 32 cases; the rest were negative in bacterial culture but all histopathologically positive with inflammatory change. Relatively high positive rate is attributed to the adequacy and accuracy of sample extraction from the infected nucleus and endplates under endoscopic view. Our results therefore correlate well with the observations reported above.

Percutaneous spine endoscope-assisted surgery minimizes surgical risk because it leaves the posterior structures undamaged and bypasses the dural sac, nerve roots, and other important structures inside the canal. Endoscopic vision allows sample collection for bacterial culture and pathological analysis, and assists the thorough lavage of the foyer with abundant normal saline until a well-debrided view of the disc surfaces. Destabilizing destruction of the vertebral bodies due to infection can lead to deformity and requires dorsal internal fixation to correct kyphosis [1, 24]. Radical debridement and systemic antibiotic use effectively controlled the progression of infection-induced bony destruction. With extra instrumentation, early mobilization and reduction of kyphotic progress can be achieved. Thus, PED plus PPST proves to be advantageous for the multicomorbid patients with advanced age as it results in little trauma, less blood loss, less intra-operative spread of infection, and faster neurological and functional recovery. Under endoscopic vision, the infected focus manages to be largely extirpated and then sent for microbiological confirmation. The instrumentation enhances vertebral stability and rapidly reduces back pain and helps achieve early mobilization, shorter hospital stay, and rapid fusion with meeting patient satisfaction for aesthetic demands through small wound and deformity prevention.

## Conclusion

PED combined with PPST for lumbar pyogenic spondylodiscitis provides effective decompression, clearance, and drainage of the affected disc space as well as ameliorates lumbar stability. Furthermore, important intraspinal structures are avoided, and a high rate of identifying the causative pathogen can be achieved. We believe that it offers a valid option in addressing intervertebral space infection, as it is minimally invasive, shortens hospital stay, and avoids prolonged bed rest with an optimistic therapeutic outcome. However, its efficacy needs to be further tested due to the lack of control group and abundant samples in our study.

## References

[1] Gouliouris T., Aliyu S. H., Brown N. M. (2010). Spondylodiscitis: update on diagnosis and management. Journal of Antimicrobial Chemotherapy.

[2] Makins GH, Abbott FC (1896). On acute primary osteomyelitis of the vertebrae. Ann Surg.

[3] Tyagi R (2016). Spinal infections in children: a review. J Orthop.

[4] Nickerson Emma K., Sinha Rohitashwa (2016). Vertebral osteomyelitis in adults: an update. British Medical Bulletin.

[5] Hadjipavlou Alexander G., Mader Jon T., Necessary Jeff T., Muffoletto Anthony J. (2000). Hematogenous Pyogenic Spinal Infections and Their Surgical Management. Spine.

[6] Gonzalvo Augusto, Abdulla Irfan, Riazi Arash, De La Harpe David (2011). Single-level/Single-stage Debridement and Posterior Instrumented Fusion in the Treatment of Spontaneous Pyogenic Osteomyelitis/Discitis. Journal of Spinal Disorders & Techniques.

[7] Pee Yong Hun, Park Jong Dae, Choi Young-Geun, Lee Sang-Ho (2008). Anterior debridement and fusion followed by posterior pedicle screw fixation in pyogenic spondylodiscitis: autologous iliac bone strut versus cage. Journal of Neurosurgery: Spine.

[8] Guerado E, Cervan AM (2012). Surgical treatment of spondylodiscitis. An update[J]. Int Orthop.

[9] Tschöke S K, Fuchs H, Schmidt O, et al(2015)Single-stage debridement and spinal fusion using PEEK cages through a posterior approach for eradication of lumbar pyogenic spondylodiscitis: a safe treatment strategy for a detrimental condition. Patient Safety in Surgery. 9(1): 35. 10.1186/s13037-015-0083-4 10.1186/s13037-015-0083-4 PMC4641346 26561500

[10] Akiyama Toru, Chikuda Hirotaka, Yasunaga Hideo, Horiguchi Hiromasa, Fushimi Kiyohide, Saita Kazuo (2013). Incidence and risk factors for mortality of vertebral osteomyelitis: a retrospective analysis using the Japanese diagnosis procedure combination database. BMJ Open.

[11] Yang Shih-Chieh, Fu Tsai-Sheng, Chen Lih-Huei, Niu Chi-Chien, Lai Po-Liang, Chen Wen-Jer (2006). Percutaneous endoscopic discectomy and drainage for infectious spondylitis. International Orthopaedics.

[12] Yang Shih-Chieh, Chen Wen-Jer, Chen Hung-Shu, Kao Yu-Hsien, Yu Shang-Won, Tu Yuan-Kun (2014). Extended indications of percutaneous endoscopic lavage and drainage for the treatment of lumbar infectious spondylitis. European Spine Journal.

[13] Reito Aleksi, Kyrölä Kati, Pekkanen Liisa, Paloneva Juha (2018). Specific spinal pathologies in adult patients with an acute or subacute atraumatic low back pain in the emergency department. International Orthopaedics.

[14] Pintado-García Vicente (2008). Espondilitis infecciosa. Enfermedades Infecciosas y Microbiología Clínica.

[15] Bairy M., Sett A., Bhandari S., Long E. (2008). Obstruction or renal allograft rejection--potential clinical markers of BK virus nephropathy. QJM.

[16] Shetty Ajoy Prasad, Aiyer Siddharth N., Kanna Rishi Mugesh, Maheswaran Anupama, Rajasekaran Shanmuganathan (2015). Pyogenic lumbar spondylodiscitis treated with transforaminal lumbar interbody fusion: safety and outcomes. International Orthopaedics.

[17] Nasto Luigi A., Colangelo Debora, Mazzotta Valentina, Di Meco Eugenia, Neri Valentina, Nasto Riccardo A., Fantoni Massimo, Pola Enrico (2014). Is posterior percutaneous screw-rod instrumentation a safe and effective alternative approach to TLSO rigid bracing for single-level pyogenic spondylodiscitis? Results of a retrospective cohort analysis. The Spine Journal.

[18] Deininger Martin H., Unfried Miriam I., Vougioukas Vassilios I., Hubbe Ulrich (2009). Minimally invasive dorsal percutaneous spondylodesis for the treatment of adult pyogenic spondylodiscitis. Acta Neurochirurgica.

[19] Mobbs RJ, Sivabalan P, Li J (2011). Technique, challenges and indications for percutaneous pedicle screw fixation. J Clin Neurosci.

[20] Lin T, Tsai T, Lu M (2014). Comparison of two-stage open versus percutaneous pedicle screw fixation in treating pyogenic spondylodiscitis[J]. BMC Musculoskelet Disord.

[22] Rankine J J (2004). Therapeutic impact of percutaneous spinal biopsy in spinal infection. Postgraduate Medical Journal.

[23] Staatz G, Adam G B, Keulers P, Vorwerk D, Günther R W (1998). Spondylodiskitic abscesses: CT-guided percutaneous catheter drainage. Radiology.

[24] FOUQUET B, GOUPILLE P, JATTIOT F, COTTY P, LAPIERRE F, VALAT J P, AMOUROUX J, BENATRE A (1992). Discitis After Lumbar Disc Surgery. Spine.

[25] Homagk L., Homagk N., Klauss J. R., Roehl K., Hofmann G. O., Marmelstein D. (2015). Spondylodiscitis severity code: scoring system for the classification and treatment of non-specific spondylodiscitis. European Spine Journal.

